# Who Wins the Battle Against Obesity? A Network Meta‐Analysis Comparing Tirzepatide and Semaglutide

**DOI:** 10.1111/1753-0407.70192

**Published:** 2026-02-10

**Authors:** Julia C. Bernardi, Deivyd V. S. Cavalcante, Ramon Huntermann, Maria E. Molinari, Luana Z. Zanon, Jacinthe Khater, Victor A. Gomez, Caroline O. Fischer‐Bacca

**Affiliations:** ^1^ University Center for the Development of Alto Vale Rio do Sul Brazil; ^2^ University of North Texas Health Science Center USA; ^3^ Faculty of Medical Sciences Lebanese University Hadath Lebanon; ^4^ Universidad de Iberoamérica (UNIBE) San Jose Costa Rica

**Keywords:** obese patients, reduction of body weight, semaglutide, tirzepatide

## Abstract

**Introduction:**

Pharmacological therapies are recommended for individuals with obesity. Semaglutide, a glucagon‐like peptide‐1 receptor agonist (GLP‐1), and tirzepatide, a dual glucose‐dependent insulinotropic polypeptide and GLP‐1 receptor agonist (GIP/GLP‐1), are among the leading pharmacological options for obesity treatment. This network meta‐analysis (NMA) aims to evaluate the comparative efficacy of these two agents in reducing body weight and improving glycemic parameters.

**Methods:**

Pairwise comparisons within the NMA were conducted using a frequentist approach in RCTs comparing tirzepatide or semaglutide versus placebo, as well as tirzepatide versus semaglutide at their maximum dosages (15 and 2.4 mg, respectively).

**Results:**

A total of 28 RCTs were included, comprising 34 367 participants, 39.6% of whom were women and with a mean age of 57.8 ± 8.95 years. Tirzepatide demonstrated superiority over semaglutide in percentage weight reduction (mean difference [MD] 6.10%; 95% CI: 3.64, 8.57), absolute weight loss (MD 4.55 kg; 95% CI: 1.28, 7.83), BMI reduction (MD 1.71 kg/m^2^; 95% CI: 0.08, 3.34), and waist circumference reduction (MD 2.89 cm; 95% CI: 1.25, 4.53). Regarding glycemic parameters, tirzepatide was also more effective than semaglutide in reducing HbA1c (MD 0.33%; 95% CI: 0.20, 0.46) and fasting blood glucose (MD 10.39 mg/dL; 95% CI: 4.48, 16.29).

**Conclusion:**

In this NMA, tirzepatide appeared to be superior to semaglutide in both body weight reduction and improvement of glycemic indices.

## Highlights

Tirzepatide demonstrated greater effectiveness in reducing body weight compared to semaglutide and placebo, both in percentage and absolute kilograms, and also led to more pronounced reductions in BMI and waist circumference.

Beyond weight loss, tirzepatide was superior to semaglutide in improving glycemic parameters, achieving greater reductions in HbA1c and fasting blood glucose in individuals with overweight or obesity.

## Graphical abstract

Network meta‐analysis comparing the effects of tirzepatide, semaglutide, and placebo on body weight reduction and glycemic outcomes in adults with overweight or obesity.

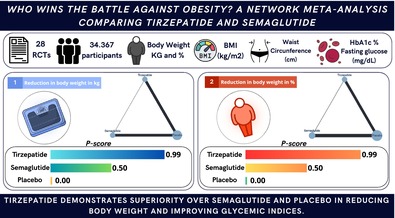

**Abbreviations**
BMI: body mass index
CI: confidence interval
FDA: Food and Drug Administration
GIP: gastric inhibitory peptide
GLP‐1: glucagon‐like peptide‐1
MD: mean difference
NMA: network meta‐analysis
OR: odds‐ratio
PICO: patient‐intervention‐comparison‐outcome
PRISMA: Preferred Reporting Items for Systematic Reviews and Meta‐Analysis
RA: receptor agonist
RCT: randomized controlled trial(s)
ROB 2: Cochrane risk‐of‐bias tool for randomized trials version 2
T2DM: type 2 diabetes mellitus

## 1 Introduction

Obesity is a chronic disease characterized by excessive fat accumulation in the body, resulting from an imbalance between caloric intake and energy expenditure [1]. It is a multifactorial condition influenced by obesogenic environments that encompass unfavorable socioeconomic and psychosocial factors [2]. The prevalence of overweight and obesity has tripled since the 1990s, negatively impacting healthcare systems [1].

The risks associated with obesity are primarily related to metabolic disorders, which contribute to the development of several diseases, such as Type 2 diabetes mellitus (T2DM), dyslipidemia, arterial hypertension, cardiovascular diseases, osteoarthritis, sleep apnea, and certain types of malignant neoplasms [2]. Weight gain is directly associated with increased cardiovascular mortality and reduced quality of life and life expectancy [2, 3].

Current therapeutic strategies recommended in clinical guidelines include lifestyle modifications, caloric intake reduction, and increased physical activity as the first‐line treatment for obesity [2, 4]. However, for individuals with a body mass index (BMI) ≥ 30 kg/m^2^, or ≥ 27 kg/m^2^ with obesity‐related comorbidities who fail to achieve adequate weight loss through lifestyle changes alone, pharmacotherapy is recommended as an adjunctive treatment [2, 4]. Glucagon‐like peptide‐1 (GLP‐1) receptor agonists have emerged as leading pharmacological options due to their favorable safety profiles and significant weight loss outcomes [2].

Semaglutide mimics endogenous GLP‐1 and activates its receptor in a prolonged manner due to chemical modifications that extend its duration of action in the body, promoting greater insulin secretion, reduced glucagon levels, delayed gastric emptying, and appetite regulation without causing hypoglycemia [5, 6]. The STEP trials demonstrated a significant and sustained weight reduction with semaglutide, along with a favorable safety and tolerability profile [5, 6, 7, 8, 9]. In 2021, the U.S. Food and Drug Administration (FDA) approved the use of 2.4 mg subcutaneous semaglutide for the treatment of obesity [10]. Subsequently, the SELECT trial demonstrated that semaglutide is effective in reducing cardiovascular events in both sexes [11].

Tirzepatide is a dual agonist of the glucose‐dependent insulinotropic polypeptide (GIP) and GLP‐1 receptors. The synergistic effect of these two incretins promotes weight loss and improves glycemic control [12]. Unlike GLP‐1, GIP regulates glucagon levels during hypoglycemic episodes and supports fat metabolism [6]. The SURMOUNT trials showed significant reductions in weight and improvements in glycemic control with a favorable safety and tolerability profile [13, 14, 15, 16]. Consequently, in 2023, tirzepatide was approved by the FDA for chronic weight management at a maximum dose of 15 mg [17].

Currently, both therapies appear promising in the management of obesity [13]. Although the SURMOUNT‐5 [18] trial represents an important advance by providing direct comparative data between the two medications, it remains a single clinical trial conducted in a specific population, which limits the generalizability of its findings. Therefore, broader analyses that integrate data from multiple studies through a network meta‐analysis (NMA) are essential to strengthen comparisons, expand the evidence base, and provide more robust estimates of the relative efficacy of these agents. This is the first NMA specifically designed to systematically compare the maximum approved doses of tirzepatide and semaglutide in individuals with overweight or obesity. This study aims to comprehensively and comparatively quantify the magnitude of tirzepatide's efficacy relative to semaglutide in promoting weight loss and improving glycemic parameters by integrating data from multiple randomized controlled trials.

## 2 Materials and Methods

This systematic review and meta‐analysis were conducted and reported in accordance with the guidelines outlined in the Cochrane Handbook for Systematic Reviews of Interventions and the Preferred Reporting Items for Systematic Reviews and Meta‐Analyses (PRISMA) statement [19, 20].

### 2.1 Eligibility Criteria

The studies included in this meta‐analysis met the following eligibility criteria: (1) randomized controlled trials (RCTs); (2) population with overweight or obesity; (3) inclusion of subjects with or without T2DM or other concomitant diagnoses; (4) direct comparison between subcutaneous tirzepatide (15 mg) and placebo; (5) direct comparison between subcutaneous semaglutide (2.4 mg) and placebo; (6) direct comparison between subcutaneous tirzepatide (15 mg) and subcutaneous semaglutide (2.4 mg); and (7) reporting of outcomes related to changes in body weight (kg and %), BMI, or waist circumference (cm). In addition, studies were included only if they reported at least one of the clinical outcomes of interest. Studies were excluded if they met any of the following criteria: (1) used another GLP‐1 receptor agonist as comparator; (2) enrolled participants under 18 years of age; or (3) did not assess the maximum approved dose of the intervention.

### 2.2 Endpoints and Sub Analyses

The primary outcomes assessed were the reduction in body weight, expressed in kilograms (kg) or percentage (%), reduction in BMI, and reduction in waist circumference (cm). Secondary outcomes included changes in glycated hemoglobin (%) and blood glucose levels (mg/dL).

### 2.3 Search Strategy and Data Extraction

A comprehensive search was conducted in PubMed, Embase, and Cochrane databases from their inception until May 2, 2025. The search strategy was constructed using the PICO framework and combined with Boolean operators (AND, OR). The following terms were used across all three databases: (“Obesity” OR “Overweight”) AND (“Weight Loss” OR “Weight Reduction”) AND (“Tirzepatide” OR “Semaglutide”). The full search strategy for each database is provided in the Supporting Information.

Additionally, the reference lists of all included studies and relevant reviews were manually screened for supplementary records. Two independent reviewers (J.C.B. and C.O.F.‐B.) screened and assessed studies according to predefined eligibility criteria and quality appraisal methods. Disagreements between reviewers were resolved through discussion and consensus, with input from a third author (R.H.). The study protocol was registered in the PROSPERO database on November 13, 2024, under registration number CRD42024614082, and was subsequently updated on June 9, 2025.

### 2.4 Statistical Analysis

Pairwise meta‐analyses and NMA were performed using a frequentist graph‐theoretical approach between November 2024 and May 2025. Continuous outcomes were pooled using mean differences (MD) with 95% confidence intervals (CI), employing the DerSimonian‐Laird estimator for between‐study variance (*τ* ^2^).

We aimed to minimize bias by including only RCTs that evaluated FDA‐approved doses of semaglutide (2.4 mg) and tirzepatide (15 mg). To assess potential heterogeneity and inconsistency across studies, we calculated the *Q* statistic, tau^2^ (*τ* ^2^), and the *I* ^2^ index. An *I* ^2^ value greater than 50% was considered indicative of moderate to high heterogeneity.

Network geometry was visualized using network plots illustrating direct comparisons and node connectivity for each outcome. The ranking of treatments was based on *P*‐scores, which reflect the extent of certainty that one intervention is superior to the others. *P*‐scores ranged from 0 (worst) to 1 (best), with higher scores indicating greater relative effectiveness.

Statistical significance was defined as a two‐sided *p* value less than 0.05. All analyses were conducted using R version 4.4.3 with the “netmeta” and “dmetar” packages for meta‐analytic modeling and diagnostic visualization. Funnel plots and Egger's test were used to evaluate publication bias, where ten or more studies were available for an outcome. Subgroup analyses were not conducted due to insufficient stratified data in the included studies.

### 2.5 Quality Assessment

The risk of bias in each study was assessed using the tool recommended by the Cochrane Collaboration Handbook [19]. For RCTs, we used the Risk of Bias in RCTs (RoB 2) tool [21]. This assessment was independently conducted by two reviewers (J.B.C. and L.Z.Z.). Any discrepancies were resolved through discussion after full‐text review, leading to a consensus among the authors. Publication bias was investigated by funnel‐plot analysis of point estimates according to study weights and by Egger's regression test [19].

The certainty of evidence for each outcome was assessed using the Grading of Recommendations, Assessment, Development and Evaluations (GRADE) system, implemented through the CINeMA (Confidence in Network Meta‐Analysis) web application [22, 23]. This tool assesses six domains: within‐study bias, reporting bias, indirection, imprecision, heterogeneity, and inconsistency to determine the overall confidence in the results of NMA.

## 3 Results

### 3.1 Study Selection and Characteristics

**FIGURE 1:**
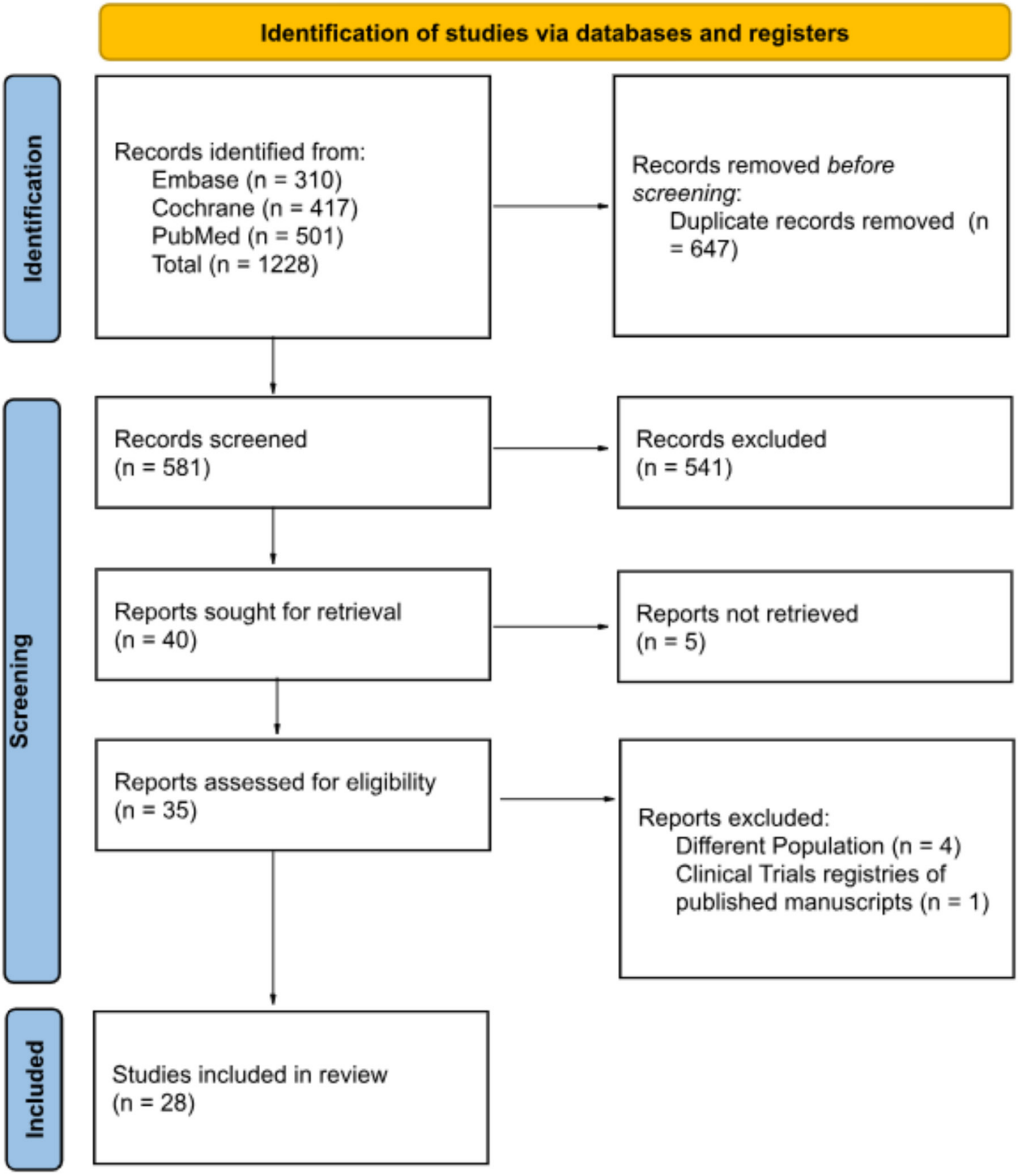
PRISMA flow diagram of study screening and selection.

As detailed in Figure 1, the initial search yielded 1228 records. After removal of duplicate records and ineligible studies, 40 remained and were fully reviewed based on inclusion criteria. Of these, a total of 28 RCTs were included, 13 studies comparing tirzepatide 15 mg with placebo [12, 13, 14, 15, 16, 24, 25, 26, 27, 28, 29, 30, 31], 14 studies comparing semaglutide 2.4 mg with placebo [5, 6, 7, 8, 9, 11, 32, 33, 34, 35, 36, 37, 38, 39], and 1 study directly comparing tirzepatide 15 mg with semaglutide 2.4 mg [18]. Figure 1 illustrates the details of the study selection process.

These studies encompassed a combined population of 34 367 participants, of whom 39.6% were female, with a mean age of 57.8 ± 8.95 years and a mean BMI of 34.2 ± 3.24 kg/m^2^. The follow‐up time between studies varied between 12 and 72 weeks. Additional study characteristics are summarized in Table 1.

**TABLE 1:** Baseline characteristics of included studies.

Study	Study design	Follow‐up^c^	Intervention	Dose^b^	Control	No of patients	Age^a^, ^d^	Female (n)	BMI^a^, ^e^	BW^f^ (kg)	WC^g^ (cm)	Hb1Ac^h^ (%)	FFG^i^ (mg/dL)
						INT/C	INT/C	INT/C	INT/C	INT/C	INT/C	INT/C	INT/C
Frias, 2018 [20]	RCT	26	TZP	15	Placebo	53/51	56.0/56.6	31/22	32.2/32.4	89.1/91.5	104.2/105.3	8.1/8.0	164.8/163.1
Frias, 2020 [27]	RCT	12	TZP	15	Placebo	28/26	56.6/56.0	5/12	31.1/32.5	86.6/89.6	105.1/109.1	8.4/8.2	194.5/168.5
STEP 1, 2021 [5]	RCT	68	SEMA	2.4	Placebo	1306/655	46/47	223/190	37.8/38.0	105.4/105.2	114.6/114.8	5.7/5.7	N/A
STEP 2, 2021 [6]	RCT	68	SEMA	2.4	Placebo	404/403	55/55	223/190	35.9/35.9	99.9/100.5	114.5/115.5	8.1/8.1	153.0/158.4
STEP 3, 2021 [7]	RCT	68	SEMA	2.4	Placebo	407/204	46/46	315/180	38.1/37.8	106.9/103.7	113.6/111.8	5.7/5.8	93.9/94.0
STEP 4, 2021 [8]	RCT	68	SEMA	2.4	Placebo	535/268	47/46	429/205	34.5/34.1	96.5/95.4	105.5/104.7	5.4/5.4	87.9/86.9
SURPASS 1, 2021 [12]	RCT	40	TZP	15	Placebo	121/115	52.9/53.6	58/59	31.5/31.7	85.4/84.9	N/A	7.85/8.05	153.3/154.8
SURPASS 5, 2022 [25]	RCT	40	TZP	15	Placebo	120/120	61/60	55/54	33.4/33.2	96.3/94.1	N/A	8.23/8.37	160.3/164.1
STEP 5, 2022 [9]	RCT	24	SEMA	2.4	Placebo	152/152	47.3/47.4	123/113	38.6/38.5	105.6/106.5	115.8/115.7	5.7/5.7	95.4/95.4
STEP 6, 2022 [32]	RCT	68	SEMA	2.4	Placebo	199/101	52/50	85/26	32.0/31.9	86.9/90.2	103.8/103.8	6.4/6.4	151.0/152.9
SURMONT‐1, 2022 [13]	RCT	72	TZP	15	Placebo	630/643	44.9/44.4	425/436	38.1/38.2	105.6/105.8	114.4/114.0	5.6/5.6	95.3/95.7
SURMONT‐2, 2023 [14]	RCT	72	TZP	15	Placebo	311/315	44.9/44.4	425/436	35.7/36.6	99.6/101.7	114.6/116.0	8.07/7.89	161.2/158.5
Heise, 2023 [28]	RCT	28	TZP	15	Placebo	45/28	61.1/60.4	14/28	31.3/32.2	94.2/98.7	113.5/109.2	7.8/7.9	139.3/126.6
SURMONT‐3, 2023 [15]	RCT	72	TZP	15	Placebo	287/292	45.5/45.7	181/183	36.1/35.7	102.5/101.3	109.3/109.6	5.3/5.4	92.6/91.3
STEP‐HEpEF, 2023 [33]	RCT	52	SEMA	2.4	Placebo	263/266	70/69	149/148	37.2/36.9	104.7/105.3	119.0/120.0	N/A	N/A
SELECT, 2023 [11]	RCT	59	SEMA	2.4	Placebo	8803/8801	61/61	2448/2424	33.3/33.4	96.5/96.8	111.3/111.4	5.78/5.78	N/A
SURMONT‐4, 2023 [16]	RCT	52	TZP	15	Placebo	335/335	49/48	236/237	30.3/30.7	84.6/85.8	96.8/98.2	5.07/5.04	85.1/85.0
STEP 7, 2024 [38]	RCT	44	SEMA	2.4	Placebo	249/126	41/40	111/59	34.0/34.0	96.4/96.2	108.5/107.0	6.1/6.3	112.2/110.8
STEP‐HEpEF, 2024 [34]	RCT	52	SEMA	2.4	Placebo	310/306	69/70	128/145	36.9/36.9	N/A	122.0/118.5	6.7/6.9	N/A
SURMONT‐CN, 2024 [30]	RCT	52	TZP	15	Placebo	71/69	35.8/37.8	35/33	32.0/32.4	91.3/92.0	104.2/105.3	5.57/5.65	92.4/92.7
STEP 10, 2024 [39]	RCT	52	SEMA	2.4	Placebo	138/69	53/53	100/47	39.9/40.4	111.9/111.0	120.1/119.9	5.9/5.9	105.1/107.7
SURMONT‐OSA, 2024 [29]	RCT	52	TZP	15	Placebo	71/69	47.3/48.9	36/41	39.7/38.6	116.7/112.8	122.6/119.8	5.69/5.64	N/A
FLOW, 2024 [35]	RCT	40	SEMA	2.4	Placebo	1767/1766	66/66	519/550	31.9/32.0	89.5/89.8	N/A	7.8/7.8	N/A
STEP 9, 2024 [36]	RCT	68	SEMA	2.4	Placebo	271/136	56/56	228/104	40.5/40.0	108.7/108.5	118.3/119.7	N/A	N/A
SUMMIT, 2025 [24]	RCT	52	TZP	15	Placebo	364/367	65.5/65.0	200/193	38.3/38.2	102.9/103.1	N/A	N/A	N/A
SURMONT‐J, 2025 [31]	RCT	72	TZP	15	Placebo	77/75	51.1/52.3	32/30	33.6/33.7	91.7/92.0	107.6/108.7	5.66/5.67	97.3/97.1
STRIDE, 2025 [37]	RCT	52	SEMA	2.4	Placebo	396/396	68/68	107/88	28.7/28.5	81.5/83.1	N/A	7.0/7.2	N/A
SURMONT‐5, 2025 [18]	RCT	72	TZP	15/2.4^j^	SEMA	374/376	45.0/44.4	242/243	39.4/39.4	112.7/113.4	117.7/118.8	N/A	N/A

Abbreviations: C: control, INT: intervention, RCT: randomized controlled trial, SEMA: semaglutide, TZP: tirzepatide.

^a^ Mean.

^b^ Dose of TZP and SEMA used in mg.

^c^ Weeks.

^d^ Years.

^e^ Body mass index (kg/m^2^).

^f^ Body weight (kg).

^g^ Waist Circumference (cm).

^h^ Glycated hemoglobin.

^i^ Fasting glucose (mg/dL).

^j^ 15 mg dose of tirzepatide and 2.4 mg dose of semaglutide.

### 3.2 Body Weight

Body weight reduction was assessed in four ways: percentage (%), kilograms (kg), BMI, and waist circumference (cm).

**FIGURE 2:**
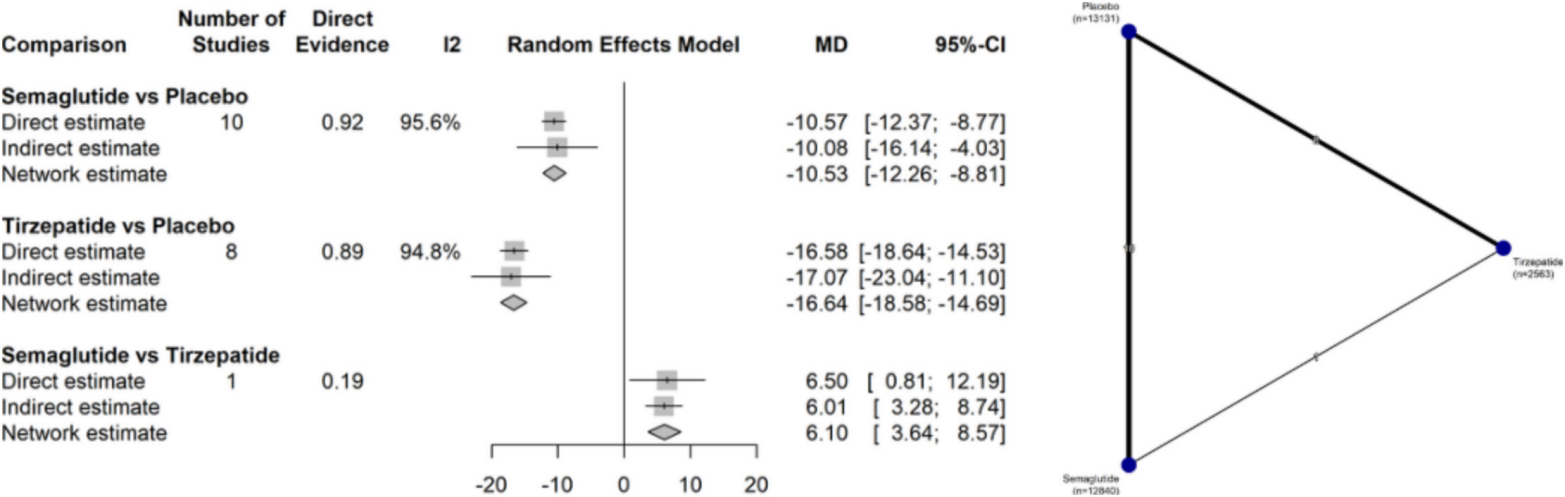
Network plot illustrating the differences between interventions and their comparators in percentage body weight reduction (%).

For percentage weight reduction, 19 studies comprising 28 424 participants were analyzed. As illustrated in Figure 2, semaglutide was superior to placebo (MD −10.53%; 95% CI: −12.26 to −8.81; *I* ^2^ = 95.6%). Tirzepatide also showed greater efficacy compared to control (MD −16.64%; 95% CI: −18.58 to −14.69; *I* ^2^ = 94.8%). In the direct comparison, tirzepatide resulted in significantly greater weight loss than semaglutide (MD 6.10%; 95% CI: 3.64 to 8.57).

**FIGURE 3:**
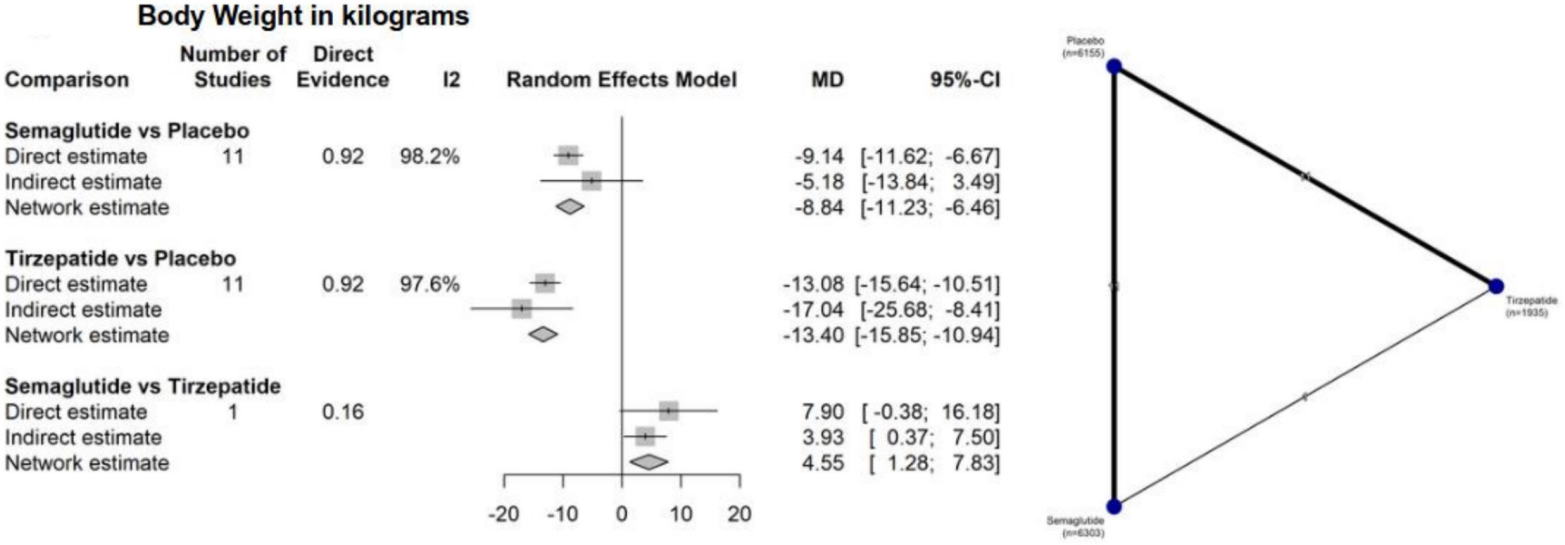
Network plot illustrating the differences between interventions and their comparators in absolute body weight reduction (kg).

Regarding absolute weight loss in kilograms, 23 studies involving 14 383 participants were analyzed. In this outcome, both interventions were superior to placebo: semaglutide versus placebo (MD −8.84 kg; 95% CI: −11.23 to −6.46; *I* ^2^ = 98.2%) and tirzepatide versus placebo (MD −13.40 kg; 95% CI: −15.85 to −10.94; *I* ^2^ = 97.6%). Tirzepatide demonstrated greater efficacy compared to semaglutide (MD 4.55 kg; 95% CI: 1.28 to 7.83) (Figure 3).

**FIGURE 4:**
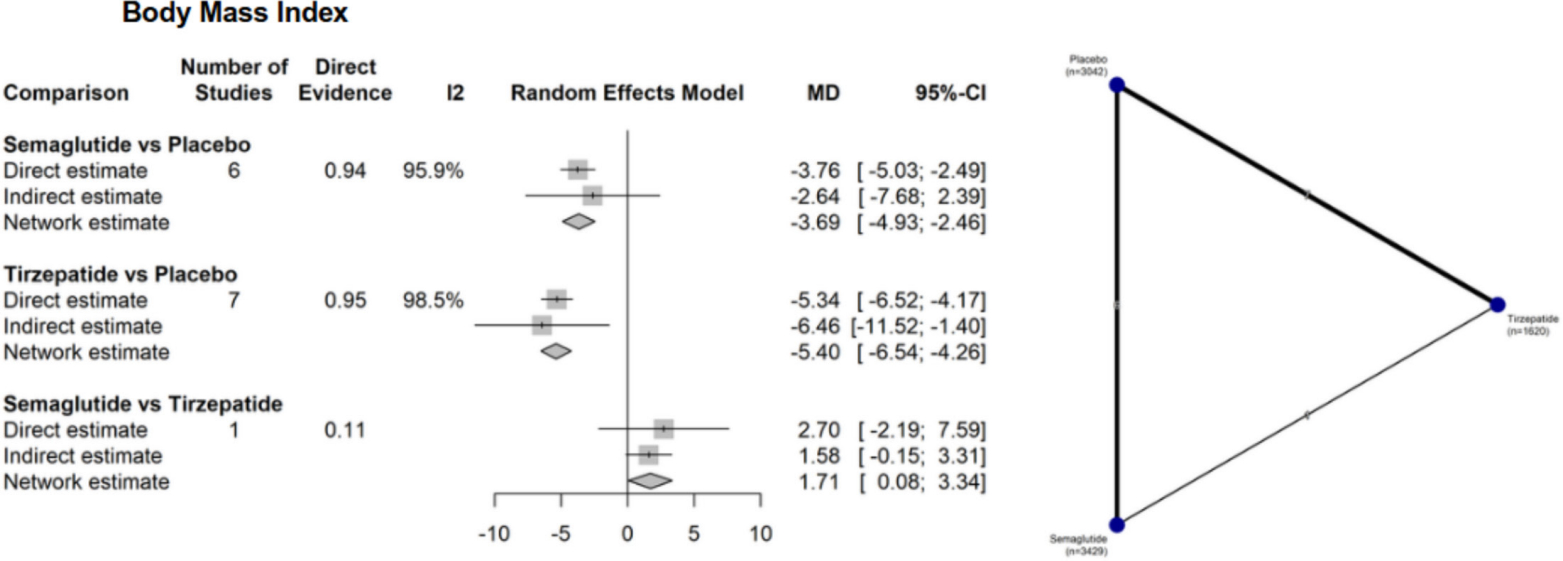
Network plot illustrating the differences between interventions and their comparators in BMI reduction.

BMI showed greater reduction with both interventions when compared to placebo: semaglutide versus placebo (MD −3.69 kg/m^2^; 95% CI: −4.93 to −2.46; *I* ^2^ = 95.9%) and tirzepatide versus placebo (MD −5.40 kg/m^2^; 95% IC: −6.54 to −4.26; *I* ^2^ = 98.5%). In the direct comparison between the two medications, tirzepatide was superior to semaglutide (MD 1.71 kg/m^2^; 95% CI: 0.08 to 3.34) (Figure 4).

**FIGURE 5:**
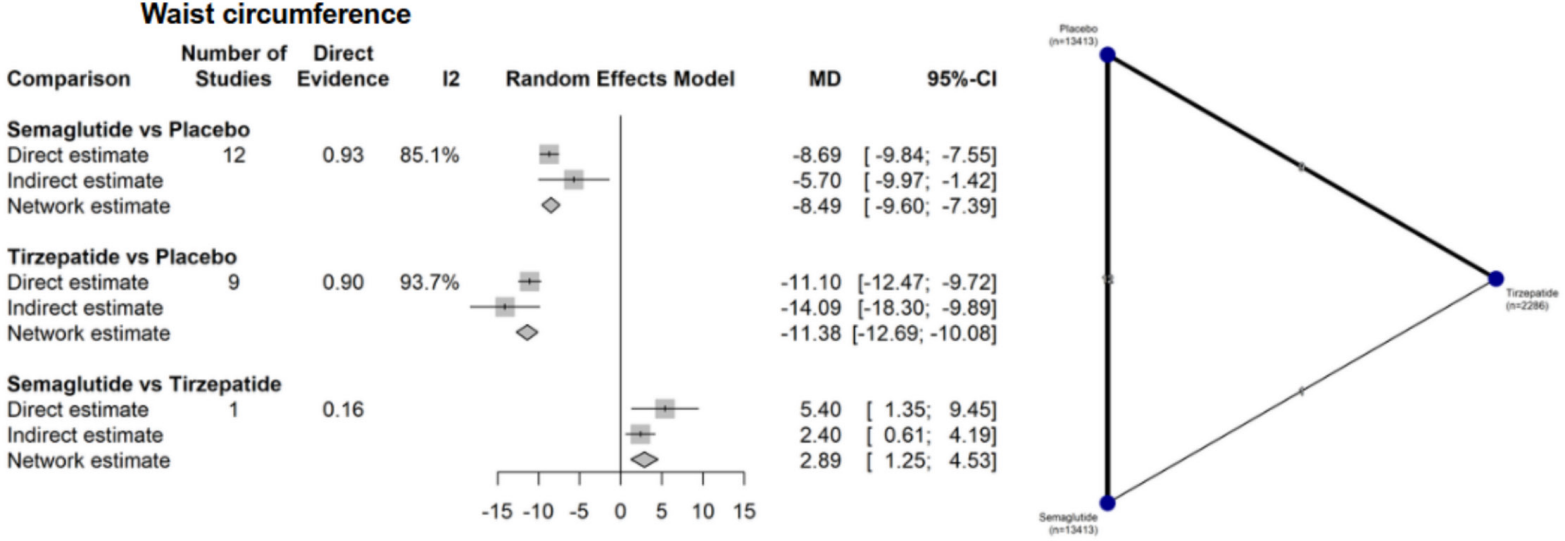
Network plot illustrating the differences between interventions and their comparators in waist circumference reduction (cm).

Similarly, when analyzing waist circumference, both interventions were superior to control: semaglutide versus placebo (MD −8.49 cm; 95% CI: −9.60 to −7.39; *I* ^2^ = 85.1%) and tirzepatide versus placebo (MD −11.38 cm; 95% CI: −12.69, −10.08; *I* ^2^ = 93.7%). In the comparison between tirzepatide and semaglutide, tirzepatide was significantly more effective in reducing waist circumference (MD 2.89 cm; 95% CI: 1.25 to 4.53) (Figure 5).

### 3.3 Glycemic Parameters

Glycemic parameters were assessed in two ways: glycated hemoglobin (HbA1c %) and blood glucose (mg/dL).

HbA1c was reported in 22 studies, encompassing 32 434 participants. In this outcome, both interventions demonstrated greater efficacy compared to placebo: semaglutide versus placebo (MD −0.57%; 95% CI: −0.65 to −0.48; *I* ^2^ = 98.1%) and tirzepatide versus placebo (MD −0.89%; 95% CI: −1.00 to −0.79; *I* ^2^ = 98.2%). Tirzepatide was more effective than semaglutide in reducing HbA1c levels (MD 0.33%; 95% CI: 0.20 to 0.46) (Figure S1). Similarly, for blood glucose reduction, both semaglutide (MD −10.07 mg/dL; 95% CI: −15.36 to −4.77; *I* ^2^ = 9.9%) and tirzepatide (MD −20.46 mg/dL; 95% CI: −24.17 to −16.74; *I* ^2^ = 96.2%) were more effective than placebo. When comparing the interventions, tirzepatide was superior to semaglutide (MD 10.39 mg/dL; 95% CI: 4.48 to 16.29) (Figure S2).

### 3.4 Ranking of Interventions

Table 2 presents the *P*‐score values, a metric used to indicate which intervention demonstrates the highest efficacy for the outcomes analyzed. The closer the *P*‐score is to 1, the greater the probability that the intervention ranks first. Tirzepatide consistently showed the best performance in percentage weight reduction (1.00), absolute weight reduction in kilograms (0.99), BMI reduction (0.99), waist circumference (0.99), HbA1c (1.00), and fasting blood glucose (0.99), indicating superior efficacy compared to semaglutide and placebo.

**TABLE 2:** P‐score for each intervention and outcome.

	BW (%)	BW (kg)	BMI (kg/m^2^)	WC (cm)	HbA1c (%)	FG (mg/dL)
Tirzepatide	**1.0**	**0.9984**	**0.99**	**0.9999**	**1.0**	**0.9999**
Semaglutide	0.5	0.5016	0.51	0.5001	0.5	0.5001
Placebo	0.0	0.000	0.00	0.0000	0.0	0.0000

*Note:* Bold values indicate the highest P‐scores, representing the intervention with the highest probability of being the most effective for the respective outcome.

Abbreviations: BMI: body mass index; BW: body weight; FG: fasting glucose; HbA1c: glycated hemoglobin; WC: waist circumference.

### 3.5 Quality Assessment

Sensitivity analyses were performed using funnel plots and Egger's test. For the outcomes of absolute weight reduction in kilograms (*p* = 0.8066), waist circumference (*p* = 0.1541), glycated hemoglobin (*p* = 0.0828), and blood glucose (*p* = 0.2920), a symmetrical distribution of studies around the mean effect was observed in the funnel plots, indicating no statistical evidence of publication bias (Figures S3–S6). For the outcome of percentage weight reduction, the results were borderline (*p* = 0.0559), suggesting a potential bias that was not statistically confirmed (Figure S7). In contrast, BMI showed statistically significant asymmetry (*p* = 0.0417), indicating possible publication bias (Figure S8).

### 3.6 Risk of Bias Assessment

The Cochrane Collaboration's tool for assessing RoB‐2 [19] was used for quality assessment. Most RCTs were judged to have a low risk of bias across the five assessed domains. The studies by Frias et al. [27] raised some concerns due to the lack of detailed information on the randomization process and the absence of a clear description of how data from participants who discontinued the study were handled. The SURMOUNT‐5 [18] was conducted as an open‐label study, which may have introduced bias in outcome assessment. The study by Heise et al. [28] was assessed as having a high risk of bias despite its randomized, double‐blind design, as it was an exploratory analysis with no imputation of missing data and no adjustment for multiplicity, limiting the reliability of its findings. The resulting figure is presented in Figure S9.

### 3.7 GRADE Assessment via CINeMA in NMA

The certainty of evidence was assessed using the CINeMA application (Confidence in Network Meta‐Analysis), a tool developed by the Cochrane group to apply the principles of GRADE to NMA [22, 23]. This approach enables a comprehensive evaluation of six core domains: within‐study bias, reporting bias, indirectness, imprecision, heterogeneity, and incoherence.

In the present study, most comparisons yielded high certainty of evidence, despite some specific methodological limitations. Outcomes such as percentage and absolute reduction in body weight, BMI, waist circumference, HbA1c, and fasting glucose were comparatively assessed between tirzepatide, semaglutide, and placebo.

Although moderate concerns regarding indirectness were identified in several comparisons, imprecision and heterogeneity did not substantially affect the overall confidence in the results. The direct comparison between semaglutide and tirzepatide, although based on a single study, maintained high certainty of evidence across all evaluated outcomes, despite some concerns related to heterogeneity, particularly for absolute weight and waist circumference.

The use of CINeMA also identified minor incoherence for the fasting glucose outcome, which did not translate into clinically meaningful impact. Overall, the consistency of findings and the absence of critical bias strengthen the robustness of the estimates.

In summary, the results of this NMA are supported by predominantly high‐certainty evidence, lending greater confidence to the conclusions, particularly regarding the superiority of the active interventions over placebo in improving the evaluated metabolic parameters. A full summary of outcomes is presented in Table S1.

## 4 Discussion

In this NMA comprising 28 RCTs and a total of 34 367 participants with obesity, the efficacy of tirzepatide was compared to that of semaglutide and placebo. The main findings included: (1) a significant reduction in percentage of body weight; (2) a considerable decrease in body weight (kg); (3) a marked reduction in BMI; (4) a decrease in waist circumference (cm); (5) significant reductions in HbA1c levels; and (6) improvements in glycemic control. These results are illustrated in the graphical abstract image, showing that all evaluated outcomes favored tirzepatide, reinforcing its therapeutic superiority over semaglutide and placebo.

The pathophysiological complexity of obesity, characterized by multifactorial mechanisms leading to an imbalance between energy intake and expenditure, responds more effectively to therapies that act simultaneously on multiple targets [1]. While semaglutide is classified as a monoagonist, acting solely on the GLP‐1 receptor, tirzepatide exerts a dual agonist effect by targeting both GLP‐1 and GIP receptors [40, 41]. This dual action engages multiple metabolic and neuroendocrine pathways, providing a more effective therapeutic response to the complexities inherent in the obese state.

Our findings are in line with previously published evidence in the literature. The SURMOUNT [13, 14, 15, 16] and STEP trials [5, 6, 7, 8, 9] evaluated incretin‐based therapies individually against placebo and reported favorable effects on weight reduction among participants. Similarly, our initial analysis also identified the superiority of both interventions over placebo across the primary outcomes assessed. Moreover, the SURMOUNT‐5 trial [18], the only study to date that directly compared tirzepatide with semaglutide in individuals with obesity, demonstrated the superiority of tirzepatide, thereby corroborating the findings observed in our indirect comparative analysis.

Currently, weight reduction in obese patients is recognized as a central factor in the effort to achieve remission or improvement of complications arising from obesity. However, non‐pharmacological interventions alone rarely lead to clinically meaningful and sustained weight loss and are generally insufficient to reach the thresholds required for remission or substantial improvement of associated comorbidities [42]. Moreover, current clinical guidelines face limitations in establishing more ambitious therapeutic goals, largely due to the difficulty in achieving significant weight reductions with traditional treatment options [2, 3]. In this context, tirzepatide and semaglutide have emerged as promising pharmacological interventions capable not only of promoting substantial weight loss but also of providing additional benefits across multiple clinical domains. Supporting evidence is found in the SURMOUNT‐OSA study [29], which evaluated patients with obstructive sleep apnea and obesity, demonstrating that those treated with tirzepatide experienced both a reduction in disease activity and an 18%–20% decrease in body weight. Similarly, the STEP‐HFpEF trials [33, 34] showed that treatment with semaglutide led to weight loss and improvements in functional capacity, cardiac biomarkers, and quality of life in patients with obesity and heart failure with preserved ejection fraction. These findings reinforce the potential of these novel therapies not only as weight‐loss agents but also as tools for the integrated management of cardiometabolic complications associated with obesity.

The growing interest in pharmacological approaches also reflects the search for less invasive alternatives to bariatric surgery. Although surgical methods are still considered the most effective interventions for achieving and maintaining substantial weight loss, yielding average reductions of up to 25% of initial body weight, with greater long‐term durability and significant improvements in metabolic conditions [43], their invasive nature, elevated risk of complications, and the need for hospitalization drive many patients to seek less aggressive, more applicable, and inherently safer alternatives. In this context, the SURPASS 1 trial [12] demonstrated that tirzepatide has a favorable safety profile, with adverse events being predominantly gastrointestinal, mild to moderate in intensity, and with few serious events reported. Combined with weight reductions approaching 20%, these findings position tirzepatide as an effective and safe therapeutic option, particularly suitable for individuals with contraindications to surgery or those who prefer less invasive approaches.

Beyond these average treatment effects, emerging evidence suggests that certain clinical subgroups may experience different magnitudes of benefit with incretin‐based therapies. Participants without Type 2 diabetes have demonstrated greater relative weight loss with tirzepatide compared with those with diabetes [13, 14], and individuals with more severe obesity often exhibit larger absolute reductions in body weight with both agents [5, 9]. Moreover, although the results achieved with the maximum doses are substantial and clinically meaningful, lower or submaximal doses have also demonstrated efficacy superior to conventional obesity treatment [44]. Considering these aspects and the reality of resource‐limited settings, it becomes evident that therapeutic decisions cannot rely exclusively on maximal efficacy. In such contexts, tirzepatide may be preferentially indicated for individuals with severe obesity, obesity‐related organ dysfunction, or Type 2 diabetes requiring more rapid and intense metabolic improvement [24, 29]. Conversely, semaglutide remains an appropriate first‐line option when cost, availability, or tolerability restricts the use of tirzepatide, particularly in patients who may still benefit from weight reductions in the range of 10%–15% [45].

This study has some limitations, primarily related to the scarcity of trials directly comparing tirzepatide and semaglutide. This limitation required the use of indirect comparisons across studies that, although sharing similar methodological characteristics and including participants with obesity, displayed variations in the clinical profiles of the evaluated populations. In particular, racial/ethnic backgrounds and the severity of comorbidities differed across trials, but the available data were insufficient to support stratified analyses; this variability may have influenced the magnitude of the therapeutic response and contributed to the heterogeneity observed. Additionally, most of the included studies had relatively short follow‐up durations, which limits a robust long‐term assessment of both sustained weight loss and remission of obesity‐related comorbidities. On the other hand, this is the first NMA to evaluate the comparative efficacy of tirzepatide and semaglutide at their maximum recommended doses, including exclusively RCTs. Outcomes beyond absolute weight loss, such as waist circumference, were also assessed due to their relevance to cardiovascular risk.

## 5 Conclusion

Our findings demonstrate the clinical advantage of tirzepatide over semaglutide and placebo in reducing body weight, both in percentage and absolute kilograms, as well as in decreasing BMI and waist circumference. However, most available trials provide only short‐ to mid‐term follow‐up, which limits conclusions regarding the long‐term sustainability of these effects. Therefore, new randomized controlled trials directly comparing both medications at FDA‐approved doses, with follow‐up periods of at least two years and conducted in comparable populations, are needed to determine the durability of weight loss, the prolonged metabolic benefits, and the long‐term safety profile.

## Author Contributions

All authors met the authorship criteria established by the International Committee of Medical Journal Editors (ICMJE) and contributed significantly to this work. **Julia C. Bernardi:** study conception, data collection and analysis, manuscript drafting, corresponding author. **Deivyd V. S. Cavalcante, Maria E. Molinari, and Ramon Huntermann:** statistical analysis, data interpretation, critical revision. **Julia C. Bernardi and Luana Z. Zanon:** data collection, methodological support, critical review. **Julia C. Bernardi:** literature review, results organization, critical review. **Maria E. Molinari:** assistance with discussion and manuscript structuring. **Caroline O. Fischer‐Bacca:** scientific supervision, final review, manuscript approval. **Victor A. Gomez:** statistical support, interpretation of findings, critical revision. **Jacinthe Khater:** final review. All authors read and approved the final version of the manuscript.

## Funding

The authors have nothing to report.

## Conflicts of Interest

The authors declare no conflicts of interest.

## Supporting information


**Figure S1:** Network plot illustrating the differences between interventions and their comparators in glycated hemoglobin (HbA1c %) reduction.
**Figure S2:** Network plot illustrating the differences between interventions and their comparators in fasting glucose (mg/dL) reduction.
**Figure S3:** Assessment of publication bias in absolute body weight (kg) reduction.
**Figure S4:** Assessment of publication bias in waist circumference (cm) reduction.
**Figure S5:** Assessment of publication bias in glycated hemoglobin (HbA1c %) reduction.
**Figure S6:** Assessment of publication bias in blood glucose (mg/dL) reduction.
**Figure S7:** Assessment of publication bias in percentage body weight (%) reduction.
**Figure S8:** Assessment of publication bias in BMI reduction.
**Figure S9:** Risk of bias assessment, Rob2.
**Table S1:** GRADE‐based assessment of evidence certainty via CINeMA for each outcome.
**Methods S1:** Search strategy.

## Data Availability

The data used in this study were extracted from previously published studies. All data supporting the findings of this study are available within the article and its Supporting Information.
