# Supplementary material for: Who Wins the Battle Against Obesity? A Network Meta‐Analysis Comparing Tirzepatide and Semaglutide
Source: J Diabetes. 2026 Feb 10;18(2):e70192. doi: 10.1111/1753-0407.70192 (PMC12887578; doi:10.1111/1753-0407.70192)
Supplement: Supplementary file 1 — Figure S1: Network plot illustrating the differences between interventions and their comparators in glycated hemoglobin (HbA1c %) reduction. Figure S2: Network plot illustrating the differences between interventions and their comparators in fasting glucose (mg/dL) reduction. Figure S3: Assessment of publication bias in absolute body weight (kg) reduction. Figure S4: Assessment of publication bias in waist circumference (cm) reduction. Figure S5: Assessment of publication bias in glycated hemoglobin (HbA1c %) reduction. Figure S6: Assessment of publication bias in blood glucose (mg/dL) reduction. Figure S7: Assessment of publication bias in percentage body weight (%) reduction. Figure S8: Assessment of publication bias in BMI reduction. Figure S9: Risk of bias assessment, Rob2. Table S1: GRADE‐based assessment of evidence certainty via CINeMA for each outcome. Methods S1: Search strategy. [file JDB-18-e70192-s001.docx]

**SUPPLEMENTAL MATERIAL**

**Table of Contents**

**Supplemental Methods 1** – Search strategy.

**Supplemental Figure 1** – Network plot illustrating the differences between interventions and their comparators in glycated hemoglobin (HbA1c %) reduction.

**Supplemental Figure 2** – Network plot illustrating the differences between interventions and their comparators in fasting glucose (mg/dL) reduction.

**Supplemental Figure** **3 –** Assessment of publication bias in absolute body weight (kg) reduction.

**Supplemental Figure 4 –** Assessment of publication bias in waist circumference (cm) reduction

**Supplemental Figure 5 –** Assessment of publication bias in glycated hemoglobin (HbA1c %) reduction

**Supplemental Figure 6 –** Assessment of publication bias in blood glucose (mg/dL) reduction

**Supplemental Figure 7 –** Assessment of publication bias in percentage body weight (%) reduction

**Supplemental Figure 8 –** Assessment of publication bias in BMI reduction

**Supplemental Figure 9** – Risk of bias assessment, Rob2

**Supplemental Table 1** – GRADE-based assessment of evidence certainty via CINeMA for each outcome

**Supplemental Methods 1. Search strategy**

Search strategy applied in PubMed, EMBASE, and Cochrane Library:

(Obesity OR Overweight) AND (Weight Loss OR Weight Reduction OR Body Weight) AND (Tirzepatide OR LY3298176 OR Mounjaro OR Zepbound OR Semaglutide OR Ozempic OR Rybelsus OR Wegovy) AND (Clinical Trial OR Trial OR Randomized Controlled Trials OR Clinical Trials, Randomized OR Trials, Randomized Clinical OR Controlled Clinical Trials, Randomized)

**Supplemental Figure 1** – Network plot illustrating the differences between interventions and their comparators in glycated hemoglobin (HbA1c %) reduction.


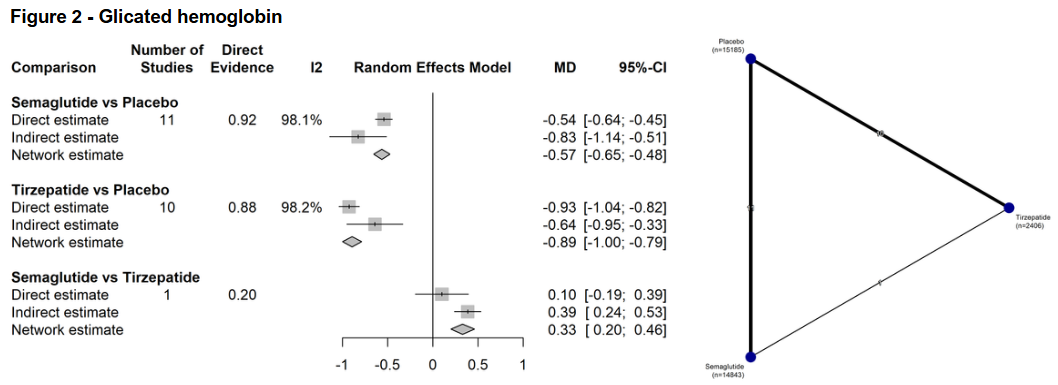


**Supplemental Figure 2** – Network plot illustrating the differences between interventions and their comparators in fasting glucose (mg/dL) reduction.


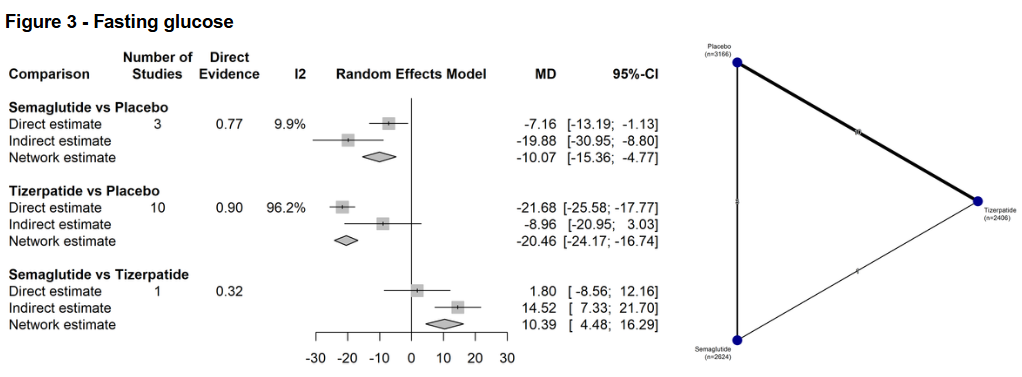


**Supplemental Figure** **3 –** Assessment of publication bias in absolute body weight (kg) reduction.


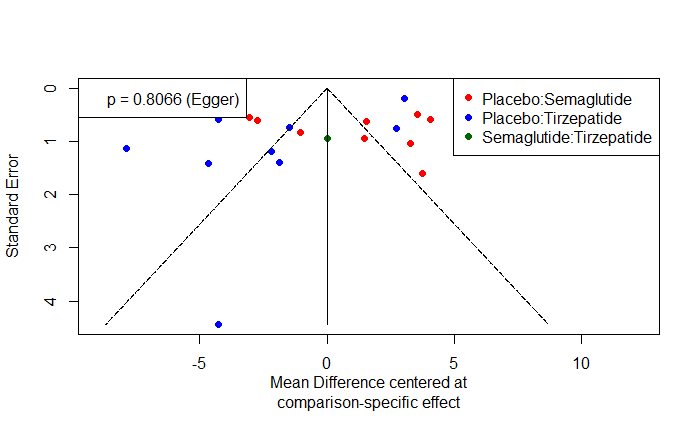


**Supplemental Figure 4 –** Assessment of publication bias in waist circumference (cm) reduction


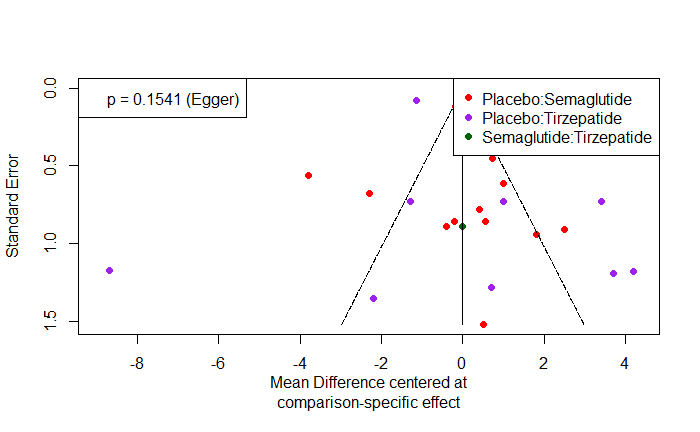


**Supplemental Figure 5 –** Assessment of publication bias in glycated hemoglobin (HbA1c %) reduction


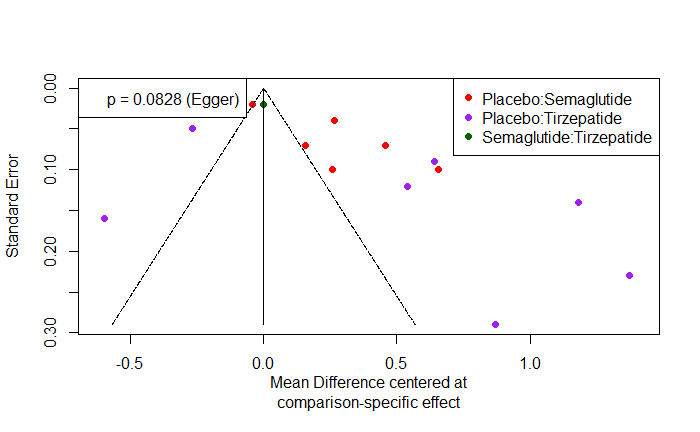


**Supplemental Figure 6 –** Assessment of publication bias in blood glucose (mg/dL) reduction


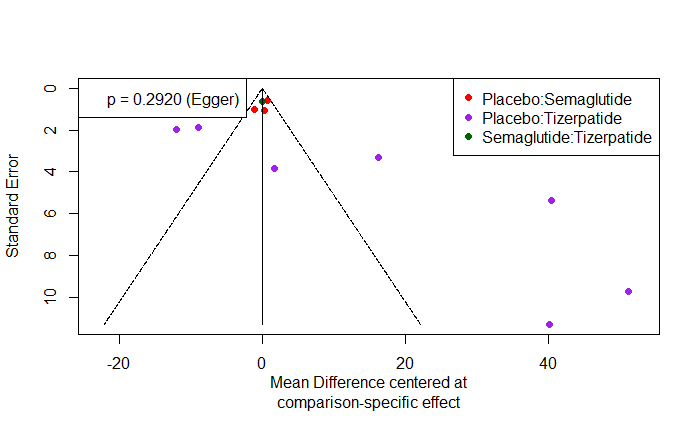


**Supplemental Figure 7 –** Assessment of publication bias in percentage body weight (%) reduction


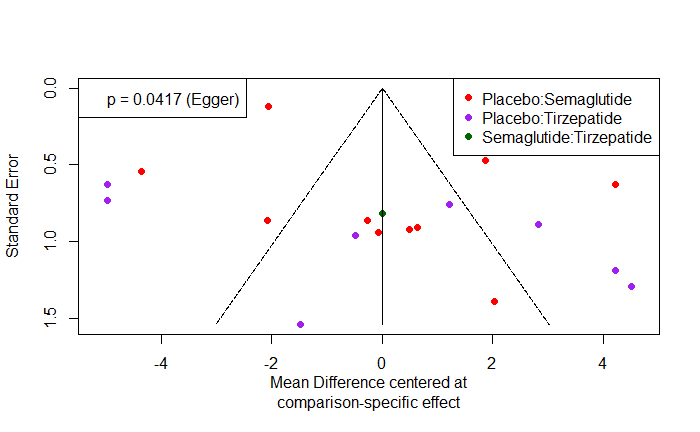


**Supplemental Figure 8 –** Assessment of publication bias in BMI reduction


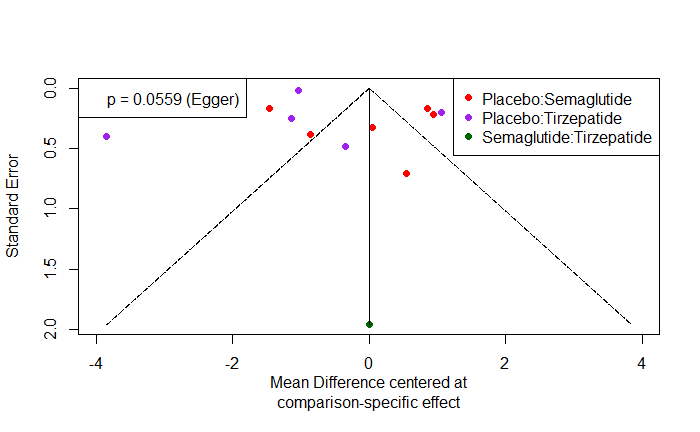


**Supplemental Figure 9** - Quality assessment with the Cochrane tool for assessing risk of bias in randomized trials (RoB 2).


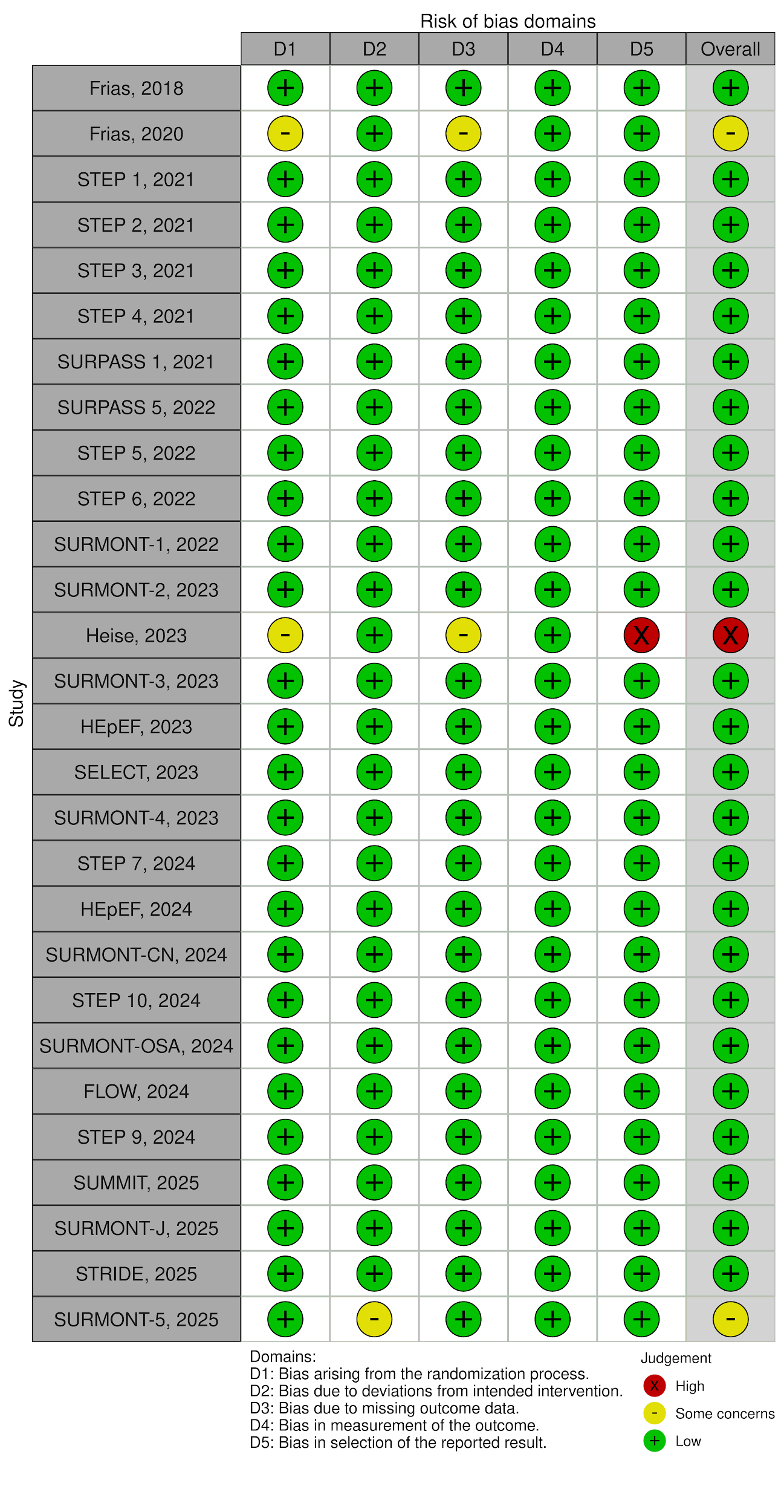


**Supplemental Table 1** - GRADE-based assessment of evidence certainty via CINeMA for each outcome

| ***Body weight (%)*** | | | | | | | | | |
| --- | --- | --- | --- | --- | --- | --- | --- | --- | --- |
| **Comparison** | **Number of studies** | **Within-study bias** | **Reporting bias** | **Indirectness** | **Imprecision** | **Heterogeneity** | **Incoherence** | **Confidence rating** | **Reason(s) for downgrading** |
| Placebo:Semaglutide | 10 | No concerns | Low risk | Some concerns | No concerns | No concerns | No concerns | High | Indirectness |
| Placebo:Tirzepatide | 8 | No concerns | Low risk | Some concerns | No concerns | No concerns | No concerns | High | Indirectness |
| Semaglutide:Tirzepatide | 1 | No concerns | Low risk | Some concerns | No concerns | Some concerns | No concerns | High | Indirectness, Heterogeneity |
| ***Body weight (kg)*** | | | | | | | | | |
| **Comparison** | **Number of studies** | **Within-study bias** | **Reporting bias** | **Indirectness** | **Imprecision** | **Heterogeneity** | **Incoherence** | **Confidence rating** | **Reason(s) for downgrading** |
| Placebo:Semaglutide | 11 | No concerns | Some concerns | Some concerns | No concerns | Some concerns | No concerns | High | Reporting bias, Indirectness, Heterogeneity |
| Placebo:Tirzepatide | 11 | No concerns | Some concerns | Some concerns | No concerns | No concerns | No concerns | High | Reporting bias, Indirectness |
| Semaglutide:Tirzepatide | 1 | No concerns | Some concerns | Some concerns | No concerns | Some concerns | No concerns | High | Reporting bias, Indirectness, Heterogeneity |
| ***BMI*** | | | | | | | | | |
| **Comparison** | **Number of studies** | **Within-study bias** | **Reporting bias** | **Indirectness** | **Imprecision** | **Heterogeneity** | **Incoherence** | **Confidence rating** | **Reason(s) for downgrading** |
| Placebo:Semaglutide | 6 | No concerns | Low risk | No concerns | No concerns | No concerns | No concerns | High | -- |
| Placebo:Tirzepatide | 7 | No concerns | Low risk | Some concerns | No concerns | No concerns | No concerns | High | Indirectness |
| Semaglutide:Tirzepatide | 1 | No concerns | Low risk | No concerns | No concerns | Major concerns | No concerns | High | Heterogeneity |
| ***Waist circumference*** | | | | | | | | | |
| **Comparison** | **Number of studies** | **Within-study bias** | **Reporting bias** | **Indirectness** | **Imprecision** | **Heterogeneity** | **Incoherence** | **Confidence rating** | **Reason(s) for downgrading** |
| Placebo:Semaglutide | 12 | No concerns | Low risk | Some concerns | No concerns | No concerns | No concerns | High | [Indirectness |
| Placebo:Tirzepatide | 9 | No concerns | Low risk | Some concerns | No concerns | No concerns | No concerns | High | Indirectness |
| Semaglutide:Tirzepatide | 1 | No concerns | Low risk | Some concerns | No concerns | Some concerns | No concerns | High | Indirectness,  Heterogeneity |
| ***HbA1c*** | | | | | | | | | |
| **Comparison** | **Number of studies** | **Within-study bias** | **Reporting bias** | **Indirectness** | **Imprecision** | **Heterogeneity** | **Incoherence** | **Confidence rating** | **Reason(s) for downgrading** |
| Placebo:Semaglutide | 11 | No concerns | Low risk | Some concerns | No concerns | No concerns | No concerns | High | Indirectness |
| Placebo:Tirzepatide | 10 | No concerns | Low risk | Some concerns | No concerns | No concerns | No concerns | High | Indirectness |
| Semaglutide:Tirzepatide | 1 | No concerns | Low risk | Some concerns | No concerns | Some concerns | No concerns | High | Indirectness, Heterogeneity |
| ***Fasting glucose*** | | | | | | | | | |
| **Comparison** | **Number of studies** | **Within-study bias** | **Reporting bias** | **Indirectness** | **Imprecision** | **Heterogeneity** | **Incoherence** | **Confidence rating** | **Reason(s) for downgrading** |
| Placebo:Semaglutide | 3 | No concerns | Low risk | No concerns | No concerns | No concerns | Some concerns | High | Incoherence |
| Placebo:Tirzepatide | 10 | No concerns | Low risk | Some concerns | No concerns | Some concerns | Some concerns | High | Indirectness, Heterogeneity, Incoherence |
| Semaglutide:Tirzepatide | 1 | No concerns | Low risk | No concerns | No concerns | No concerns | No concerns | High | -- |
